# Idiopathic scrotal hematoma in newborns: a case report and literature review

**DOI:** 10.1007/s00247-025-06357-5

**Published:** 2025-08-08

**Authors:** Carlotta Plessi, Grazia Spampinato, Enrica Caponcelli, Viviana Durante, Michelina Ciliberti, Katia Rossi, Pier Luca Ceccarelli

**Affiliations:** 1 Azienda Ospedaliero-Universitaria Policlinico di Modena, Via del Pozzo 71, 41124 Modena (MO), Italy; 2 University of Modena and Reggio Emilia, Via del Pozzo 71, 41124 Modena (MO), Italy

**Keywords:** Idiopathic scrotal hematoma, Newborn, Testicular torsion, Ultrasound

## Abstract

Neonatal acute scrotum is an uncommon condition, with testicular torsion being the most critical differential diagnosis. Idiopathic scrotal hematoma is a rare entity characterized by scrotal swelling and discoloration, with preserved testicular perfusion. Differentiating idiopathic scrotal hematoma from testicular torsion is challenging but essential to avoid unnecessary surgery. We report a case of a newborn who presented with scrotal swelling and discoloration at 72 h of life. Doppler ultrasound revealed preserved testicular blood flow, and abdominal ultrasound excluded intra-abdominal pathology. The patient was diagnosed with idiopathic scrotal hematoma and managed conservatively. A review of 16 cases of neonatal idiopathic scrotal hematoma highlights its diagnostic challenges and the potential for misdiagnosis. While early reports emphasized surgical exploration, advances in imaging, particularly Doppler ultrasound, now allow for non-invasive diagnosis and conservative management.

## Introduction

Acute scrotum in neonates is a rare but well-recognized clinical condition, with neonatal testicular torsion being the most common cause. Although the incidence of testicular torsion in this age group is relatively low, it remains a critical diagnosis due to the potential risk of testicular loss. Other conditions that can mimic testicular torsion in neonates include testicular infections and intra-abdominal processes presenting with scrotal symptoms due to a patent processus vaginalis [1].

Neonatal idiopathic scrotal hematoma is a condition in which a newborn presents with an acute scrotum, but shows normal testicular blood flow, as confirmed preoperatively by Doppler ultrasound (US) or during surgery, along with blood accumulation around the testis. The source of bleeding remains unidentified. Idiopathic scrotal hematoma is a diagnosis of exclusion and distinguishing it from testicular torsion can be particularly challenging.

In this paper, we describe a case of neonatal idiopathic scrotal hematoma observed at our clinic and provide a comprehensive review of the existing literature on this rare condition.

## Case report

**Fig. 1:**
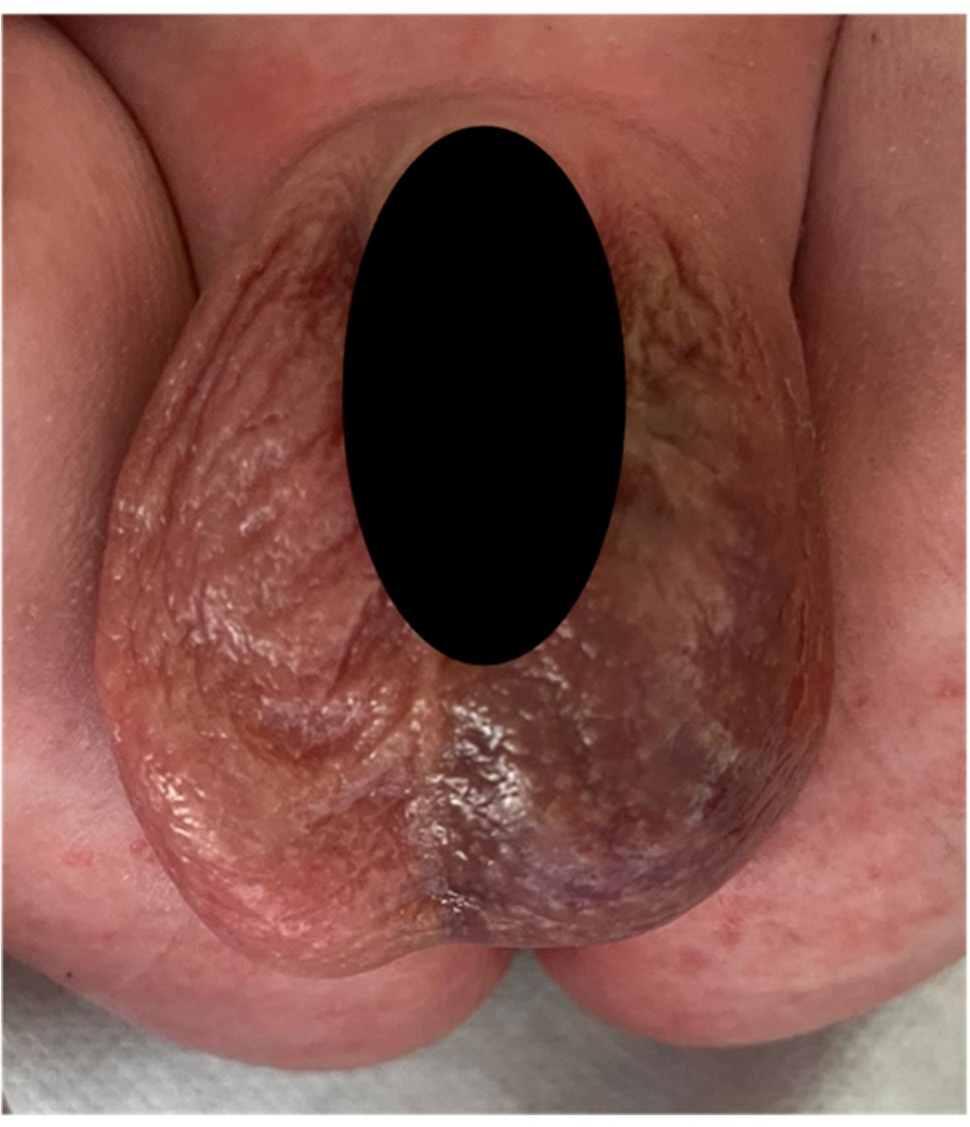
Clinical appearance of the left scrotal hematoma in a 3-day-old neonate

Informed consent was obtained from the parents for publishing the case report. A term infant (41 weeks gestational age) was delivered by a 25-year-old healthy woman after an uneventful pregnancy at our clinic. The delivery was complicated by shoulder dystocia. Birth weight was 4,200 g (95th percentile). At 72 h of life, left scrotal swelling and discoloration were noted (Fig. 1). On palpation, the left testicle was edematous, firm, and non-tender.

**Fig. 2:**
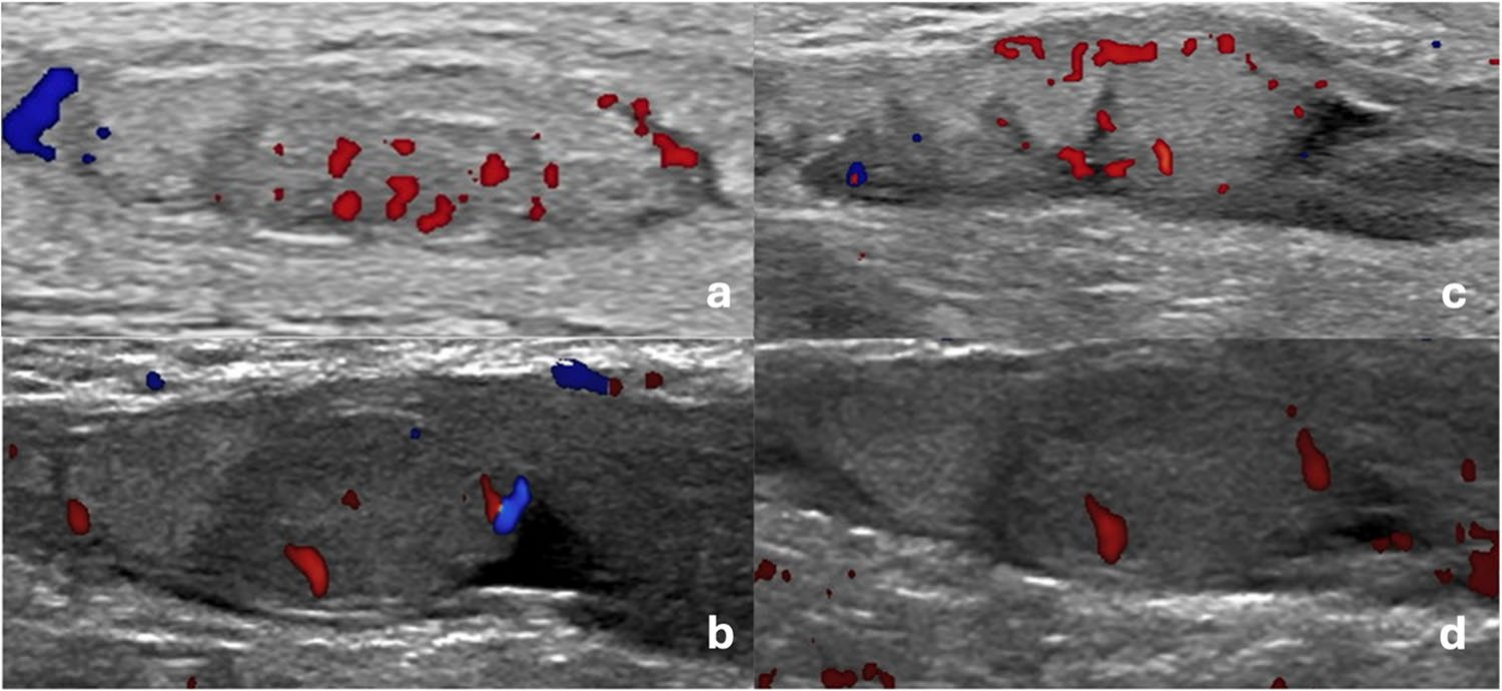
Longitudinal view of a Doppler mode ultrasound of the scrotum performed at day 3 of life, showing adequate vascularity in both testes (**a**-**b** left testicle, **c**-**d** right testicle)

Given the suspicion of neonatal testicular torsion, a testicular Doppler ultrasound (US) was performed using a Philips Affini 70 color Doppler ultrasound system (Philips, Amsterdam, Netherlands) equipped with an EL18-4 linear array transducer. The left testicle showed a normal parenchymal echo pattern and normal blood flow on color Doppler, with even increased flow compared to the contralateral testis; the testicular artery was also identified on spectral analysis; a small left hydrocele was noted (Fig. 2). Additionally, an abdominal US was carried out, which ruled out abdominal hemorrhage (particularly adrenal hemorrhages) or other pathological conditions. Back in the neonatal unit, during a further clinical examination, scrotal discoloration was still present, but palpation indicated that the testicle had become less swollen and softer. Based on the clinical evolution and the Doppler US results, the newborn was discharged with a follow-up visit scheduled in 2 days.

**Fig. 3:**
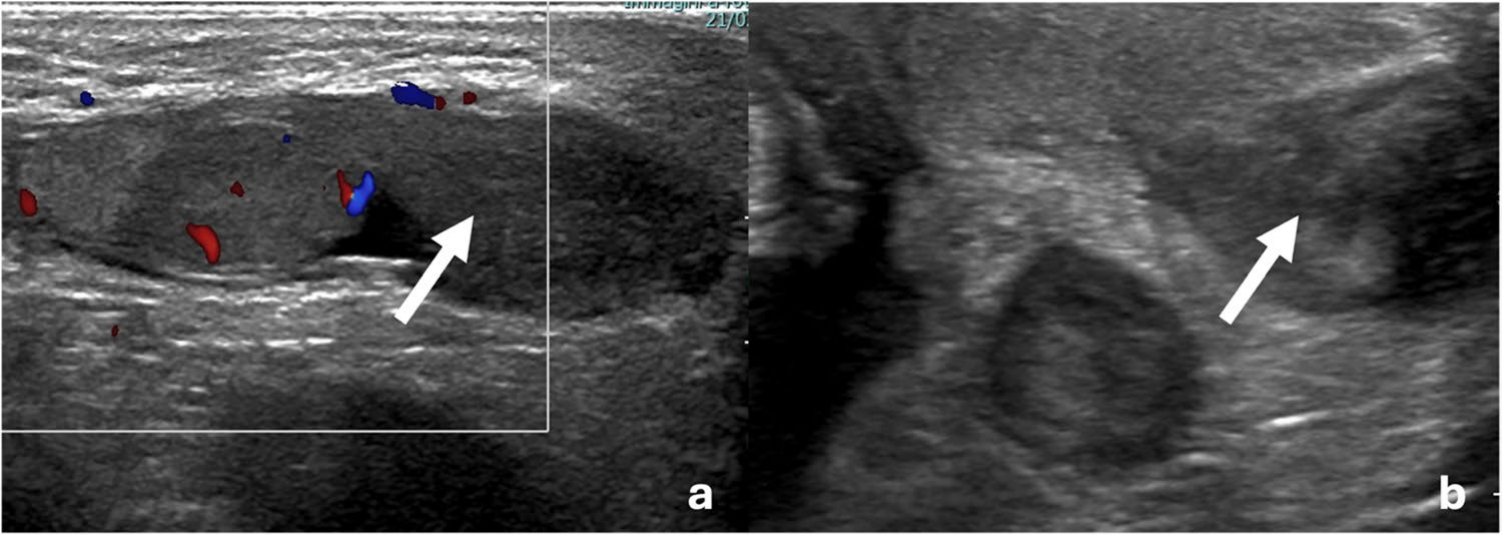
Longitudinal (**a**) and transversal (**b**) view of the testicular ultrasound performed at day 4 of life, showing the scrotal hematoma (*white arrow*)

On day 4 of life, the parents visited the emergency room, concerned about the darkening of the left hemiscrotum. A repeat testicular Doppler US and abdominal US confirmed normal testicular blood flow and revealed a scrotal hematoma (Fig. 3), with no evidence of intra-abdominal bleeding.

Based on the clinical and radiological evolution, a diagnosis of idiopathic scrotal hematoma was established. The development of the testis was normal on follow-up examinations.

## Discussion

Idiopathic scrotal hematoma is a rare but recognized condition in the neonatal population. We performed a review of the literature on this topic and found a total of 16 cases of idiopathic scrotal hematoma in neonatal age, including our case report. Clinical, radiologic, and operative information is summarized in Table 1.

**Table 1:** Review of the literature showing papers reporting cases of idiopathic scrotal hematoma in the neonatal age

Paper	No. of cases	Weight for GA (SGA/AGA/LGA)	Difficult delivery	Possible risk factors	Median age (days)	Clinical manifestation	Testicular doppler US	Abdominal US	Surgery (yes/no)	Follow-up
Davenport er al (1989) [2]	2	2/2 LGA	2/2	2/2 high birth weight and dystocia	2.5	2/2 scrotal swelling and bruising	0/2	2/2 normal	2/2	N.A
Yeh et al. (2000) [3]	4	4/4 AGA	2/4	2/4 dystocia	1	3/4 bluish discoloration of scrotum and groin 1/4 bluish discoloration of the scrotum	0/4	4/4 normal	4/4	4/4 normal
Diamond et al. (2003) [1]	5	4/5 AGA, 1/5 LGA	1/5	1/5 dystocia 2/5 bleeding diathesis	< 2	5/5 firm scrotal mass and discoloration	4/5 normal, 1/5 doubtful	4/5 normal	1/5	5/5 normal
Wu et al. (2005) [5]	1	AGA	1/1	Dystocia	1	Scrotal swelling with bluish discoloration	Suspected torsion	Normal	Yes	Normal
Jimoh et al. (2014) [7]	1	AGA	0/1	None	3	Scrotal swelling and bruising	Normal	Normal	No	Normal
Crisci et al. (2014) [8]	1	AGA	N.A	None	3	Scrotal swelling and bruising without pain	Suspected torsion	Normal	Yes	Normal
Gkantseva-Patsoura et al. (2021) [6]	1	SGA	1/1	Precipitous delivery	3	Scrotal swelling and bruising	Normal	Normal	No	Normal
Present study	1	LGA	1/1	Dystocia	2	Scrotal swelling and bruising	Normal	Normal	No	Normal

*GA* gestational age, *SGA* small for gestational age, *AGA* adequate for gestational age, *LGA* large for gestational age, *US* ultrasound

First described in 1989 by Davenport et al. [2], idiopathic scrotal hematoma was identified as a potential cause of neonatal acute scrotum and consequently as a differential diagnosis for testicular torsion. The authors suggested that an excessively high venous pressure in the scrotal veins during a difficult delivery (e.g., forceps delivery or large-for-gestational-age – LGA – newborns) could be the cause of this condition. However, in our literature review, only 3/16 patients (19%) were LGA and only 8/16 (50%) had a difficult delivery. In our case report, the initial clinical presentation strongly suggested testicular torsion, although Doppler testicular US did not confirm the diagnosis. Upon re-examination after the US, the clinical findings had changed significantly: the testicle was much softer, and persistent congestion was primarily observed in the tunica vaginalis. This could indicate intermittent testicular torsion, a condition in which symptoms spontaneously resolve, and blood flow is restored to a previously avascular testicle, as seen during US evaluation. In our opinion, the scrotal hematoma observed the following day in our patient may have been a consequence of this intermittent torsion, which likely led to severe congestion and rupture of the fragile scrotal veins, particularly vulnerable at this age.

While Davenport et al. [2] emphasized the need for surgical exploration to differentiate idiopathic scrotal hematoma from testicular torsion and to drain the hematoma, Yeh et al. [3] raised the question of whether surgery was always necessary, especially in neonatal patients with well-known anesthesia-associated risks. Acknowledging that distinguishing idiopathic scrotal hematoma from testicular torsion can be challenging, they proposed that, unlike testicular torsion, idiopathic scrotal hematoma typically does not present with discoloration in the groin, and the spermatic cord appears normal on palpation. Additionally, they suggested that imaging techniques such as radioisotope testicular scanning and Doppler US could evaluate testicular perfusion, aiding in the differentiation between idiopathic scrotal hematoma and testicular torsion.

In recent decades, significant advancements in US techniques have made it increasingly possible to avoid surgery, with diagnostic accuracy now reaching up to 92%. In addition to the absence of blood flow, indicators of testicular torsion include heterogeneity in echogenicity, thickening of the tunica albuginea, irregular margins, and the presence of hydrocele [4]. Our literature review shows that all patients reported over the past 25 years, including our case, underwent testicular Doppler US, but only three (33%) cases of torsion could not be excluded, requiring exploratory surgery. Some researchers [5, 6] also recommend abdominal US to rule out intra-abdominal causes of acute scrotum, such as intra-abdominal bleeding (particularly adrenal hemorrhage) or perforation (e.g., meconium peritonitis). In our review, abdominal US was performed in 15 out of 16 cases (94%) and was negative in all of them.

Based on a testicular doppler US negative for testicular torsion and an abdominal US negative for intra-abdominal causes of acute scrotum, a diagnosis of idiopathic scrotal hematoma can be made by exclusion. Recent studies in the literature consistently support that in such cases surgery can be avoided and a conservative approach can be safely adopted [1, 6–8]. Exceptions include compressive hematomas that compromise testicular vascularization [5] and those that become infected [7]. In all cases of conservatively treated cases of idiopathic scrotal hematoma reported in the literature, follow-up examinations have shown normal testicular development.

In conclusion, idiopathic scrotal hematoma should be considered in the differential diagnosis of acute scrotum in the neonatal period. Doppler testicular US and abdominal US are usually sufficient to distinguish idiopathic scrotal hematoma from other potential causes, particularly testicular torsion, thereby preventing unnecessary surgical exploration.

## Data Availability

No datasets were generated or analysed during the current study.
